# Zigzag carbon as efficient and stable oxygen reduction electrocatalyst for proton exchange membrane fuel cells

**DOI:** 10.1038/s41467-018-06279-x

**Published:** 2018-09-19

**Authors:** Longfei Xue, Yongcheng Li, Xiaofang Liu, Qingtao Liu, Jiaxiang Shang, Huiping Duan, Liming Dai, Jianglan Shui

**Affiliations:** 1 School of Materials Science and Engineering, Beihang University, No. 37 Xueyuan Road, Beijing, 100083 China; 2 Department of Macromolecular Science and Engineering, Case Western Reserve University, 10900 Euclid Avenue, Cleveland, OH 44106 USA; 3 UNSW-CWRU International Joint Laboratory, School of Chemical Engineering, University of New South Wales, Sydney, NSW, 2051 Australia

## Abstract

Non-precious-metal or metal-free catalysts with stability are desirable but challenging for proton exchange membrane fuel cells. Here we partially unzip a multiwall carbon nanotube to synthesize zigzag-edged graphene nanoribbons with a carbon nanotube backbone for electrocatalysis of oxygen reduction in proton exchange membrane fuel cells. Zigzag carbon exhibits a peak areal power density of 0.161 W cm^−2^ and a peak mass power density of 520 W g^−1^, superior to most non-precious-metal electrocatalysts. Notably, the stability of zigzag carbon is improved in comparison with a representative iron-nitrogen-carbon catalyst in a fuel cell with hydrogen/oxygen gases at 0.5 V. Density functional theory calculation coupled with experimentation reveal that a zigzag carbon atom is the most active site for oxygen reduction among several types of carbon defects on graphene nanoribbons in acid electrolyte. This work demonstrates that zigzag carbon is a promising electrocatalyst for low-cost and durable proton exchange membrane fuel cells.

## Abstract (web summary)

Cost and stability of catalysts hinder widespread use of proton exchange membrane fuel cells. Here the authors synthesize zigzag-edged graphene nanoribbons for electrocatalysis of oxygen reduction. Employment of such a metal-free catalyst in a fuel cell yields remarkable power density and durability.

## Introduction

Proton exchange membrane fuel cells (PEMFCs) attract worldwide attention because they can efficiently convert hydrogen energy into electricity. The device uses an acid solid electrolyte (sulfonated tetrafluoroethylene-based fluoropolymer-copolymer) and is the most mature type of fuel cell. In the PEMFC, the oxygen reduction reaction (ORR) is the rate-determining step. Noble metal platinum is generally employed as a catalyst, and has hindered the wide application of PEMFCs. To solve this problem, a variety of non-precious metal catalysts (NPMCs), including nitrogen-coordinated transition metals (M-N-C, M = Fe, Co, etc.)^1–7^, and even metal-free catalysts (mainly heteroatom-doped carbon nanomaterials) have been developed^8–12^. The NPMCs present satisfactory activity and stability during half-cell characterization (a three-electrode system) with both alkaline and acidic electrolytes. However, poor stability in the actual PEMFC device is still one of the biggest challenges for NPMCs, especially for M-N-C electrocatalysts^13–15^. Recently, an N-doped graphene/carbon nanotube (CNT) composite has been reported to exhibit a stable PEMFC performance, though at a relatively low activity, which attracted a great deal of interest in metal-free electrocatalysts for the PEMFCs^16^.

In addition to the heteroatom-doped carbon nanomaterials, recent studies demonstrated that the defect-rich carbon could also effectively catalyze ORR in both alkaline and acidic electrolytes^17–21^. Thus, graphene nanoribbons (GNRs) could be promising metal-free catalysts due to the large aspect ratio and numerous defects along their edges^22,23^. GNRs with different edge structures can be obtained by unzipping CNTs in different ways^24–27^. In particular, Tour et al. created a zigzag-type edge on unzipped multiwall carbon nanotubes (MWCNTs) using H_2_SO_4_ and KMnO_4_^25,28^. This offers an opportunity to study the specific activity of a certain type of carbon defects. In spite of the abundant defect sites, GNRs have not been characterized with any decent ORR activities in the acid half cell. The easy restacking of GNRs could lead to low activity since most of the GNR edges in the restacked materials are blocked from the reactants, leading to extremely low utilization of the active sites. This blocking effect will be more serious in a PEMFC because the catalyst layer in a PEMFC is much thicker than that in a half cell. Furthermore, in contrast to the liquid electrolyte in a half cell, the solid ionomer electrolyte in PEMFCs causes a severe mass transport barrier. So far, the promise of GNRs (or graphitic carbon defects) for electrocatalysis in PEMFCs is yet to be realized.

Here a composite of zigzag-edged GNRs on CNTs (GNR@CNT) is synthesized for use as the cathode catalyst in H_2_/O_2_ PEMFCs. The CNT backbones in the middle of GNR, coupled with a carbon black spacer, effectively exposes the zigzag carbon for ORR. Compared with previously reported metal-free electrocatalysts^16^, GNR@CNT delivers an unprecedented peak power density of 520 W g^−1^ in PEMFCs, even better than that of the N-doped counterpart. More importantly, CNT@GNR presents a remarkable stability in PEMFCs. A density functional theory (DFT) calculation reveals zigzag carbon is indeed the active site on the GNR.

## Results

### Fabrication and characterization of catalysts

**Fig. 1:**
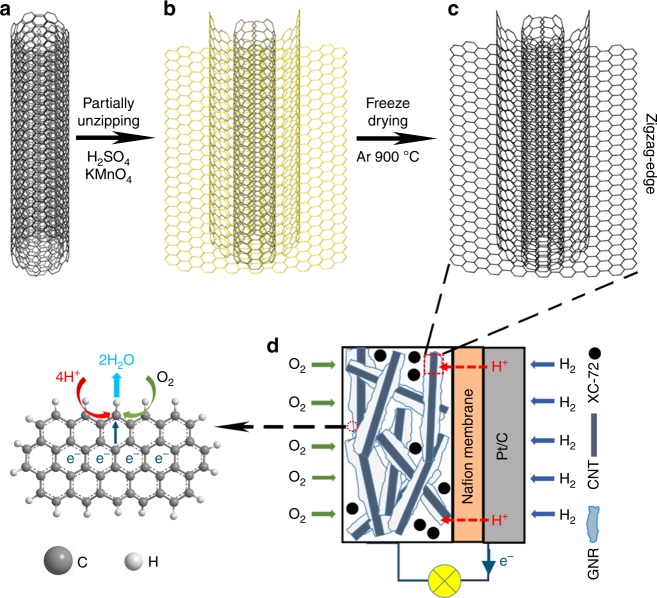
Schematic illustration. The synthetic route of zigzag-type graphene nanoribbons on carbon nanotubes (GNR@CNT) from **a** MWCNT to **b** partially unzipped oxidized CNT and to **c** GNR@CNT. **d** The application as oxygen reduction reaction catalyst in a proton exchange membrane fuel cell (PEMFC). Carbon black XC-72 is used as spacer to prevent the stacking of active materials

Figure 1 schematically illustrates the partial unzipping of a MWCNT into zigzag-edged GNR with a CNT backbone. Following the published method^25^, purified commercial MWCNTs (diameters ~20 nm and lengths of 0.5–2 μm) were partially unzipped by concentrated sulfuric acid and potassium permanganate, forming an oxidized GNR@CNT hybrid (Fig. 1b). As shown in Supplementary Fig. 1a, b, the oxidized GNR has a width of approximately 60 nm and is attached on the CNT backbone. Finally, the oxidized GNR was reduced at high temperature, producing a GNR@CNT composite. According to previous reports^25,28^, this unzipping method created zigzag-type carbon along GNR edges. GNR@CNT thus obtained was then used as the cathode catalyst in a PEMFC. The rigid CNT backbone could prevent the stacking of GNR in the catalyst layer, as schematically shown in Fig. 1d. Meanwhile, carbon black was introduced into the catalyst layer to further separate GNR@CNT, facilitating the mass transport in the catalyst layer to ensure a high utilization of zigzag carbon defects on the GNR edges^16^. As a result, GNR@CNT exhibited efficient and stable ORR catalytic activity in PEMFCs, as discussed below. For comparison, three control samples have been prepared, including a completely unzipped MWCNT (denoted GNR) using a double-concentrated KMnO_4_ than that for GNR@CNT, and the N-doped counterparts (N-GNR@CNT and N-GNR) by annealing corresponding materials in NH_3_ at 800 °C^29^.

**Fig. 2:**
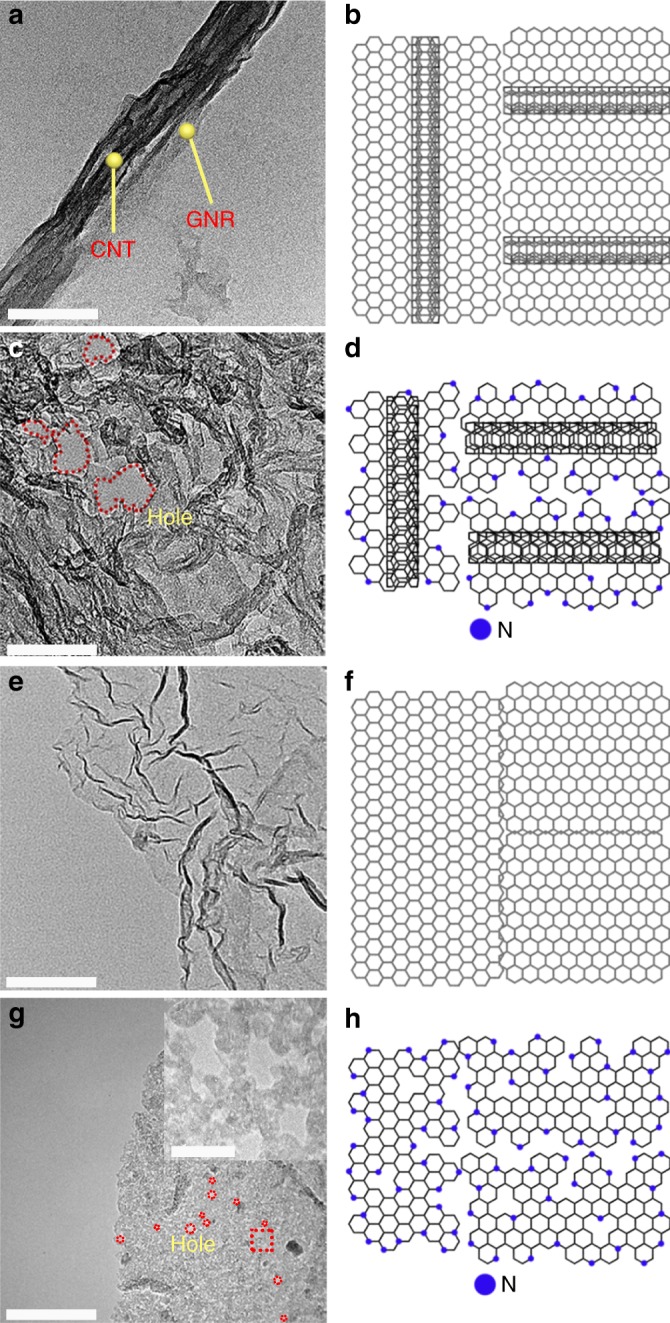
Transmission electron microscopy images and schematic diagrams. **a**, **b** Partially unzipping multiwall carbon nanotube (MWCNT) to graphene nanoribbons on carbon nanotube (GNR@CNT), **c**, **d** nitrogen-doped GNR@CNT (N-GNR@CNT), **e**, **f** totally unzipped MWCNT to graphene nanoribbons (GNR), and **g**, **h** nitrogen-doped GNR (N-GNR). Scale bar: 100 nm, and 10 nm for inset micrograph in **g**

Figure 2 and Supplementary Fig. 2 compared the morphology of GNR@CNT, GNR, and their N-doped counterparts. As shown in Fig. 2a and Supplementary Fig. 2a, the partially unzipped CNTs formed ribbon-like graphene nanosheets closely attached on the remaining CNT backbones. NH_3_-treatment could severely corrode the GNR, leaving the CNT skeleton as the dominant material, as observed in Fig. 2c and Supplementary Fig. 2b. Fourier transform infrared (FT-IR) spectra of GNR@CNT and N-GNR@CNT suggested the reduction of oxidized GNR after Ar and NH_3_ annealing (Supplementary Fig. 3), showing the removal of oxygen functional groups. Different from partially unzipped CNTs, the fully unzipped CNTs displayed a typical graphene-like morphology without any residual CNTs (Fig. 2e and Supplementary Fig. 2c). Meanwhile, NH_3_ corrosion created dense holes with diameter of 5–10 nm on N-GNR sheets (Fig. 2g and Supplementary Fig. 2d). The surface composition of GNR@CNT and N-GNR@CNT were analyzed by X-ray photoelectron spectroscopy (XPS). The survey scan did not detect any Ni signals (catalyst for the CNT growth) after the acid pretreatment (Supplementary Fig. 4a). The high-resolution C 1*s* spectrum in Supplementary Fig. 4b reveals the dominance of *sp*^2^ carbon, suggesting a highly graphitic structure of GNR@CNT. The XPS spectra of N-GNR@CNT indicates a successful doping of 3.09 at.% N, which was composed of pyridinic N (398.5 eV), pyrrolic N (399.8 eV), quaternary N (401.2 eV), and intercalated nitrogen molecules and/or oxides (403.5 eV) (Supplementary Fig. 5).

### Electrochemical performance evaluation

**Fig. 3:**
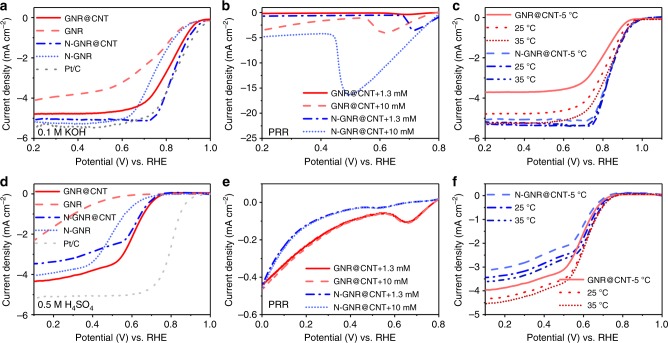
Half-cell characterization of the catalysts. Linear sweep voltammetry curves of graphene nanoribbons on carbon nanotubes (GNR@CNT), N-doped GNR@CNT (N-GNR@CNT), and N-doped graphene nanoribbons (N-GNR) for **a** oxygen reduction reaction (ORR) activity, **b** peroxide reduction reaction (PRR) activity with 1.3 or 10 mM H_2_O_2_, and **c** ORR activity at 5, 25, and 35 °C in 0.1 M KOH; **d** ORR activity, **e** PRR activity with 1.3 or 10 mM H_2_O_2_, and **f** ORR activity at 5, 25, and 35 °C in 0.5 M H_2_SO_4_. Electrolyte was O_2_-saturated, except for PRR experiments with Ar-saturated electrolyte. Rotating speed: 1600 rpm. Scan rate: 10 mV s^−1^

The electrocatalytic activity of the resultant catalysts was first thoroughly investigated in a half cell. The optimal annealing temperatures of 900 °C for GNR@CNT in Ar and 800 °C for N-GNR@CNT in NH_3_ were deduced by comparing the linear sweep voltammetry (LSV) curves in Supplementary Figs. 6, 7, respectively. Catalysts obtained under the optimized conditions were used for subsequent characterization. As shown in the above transmission electron microscopy (TEM) characterizations, ammonia treatment created an abundance of nanoholes, defects, and N-dopants in GNRs, which can facilitate mass transfer in the stacked catalyst layer and introduce significant pseudocapacitance^30,31^. Moreover, CNT backbones also improved the capacitances of GNR@CNT and N-GNR@CNT, compared with GNR and N-GNR, by alleviating stacking of GNRs (Supplementary Fig. 8)^32,33^. Benefitting from the ammonia treatment and CNT backbones, N-doped catalysts presented higher ORR activities than the non-doped counterparts; GNR@CNT and N-GNR@CNT also exhibited superior ORR activities in comparison to GNR and N-GNR counterparts in an alkaline electrolyte (Fig. 3a). It is worth noting that GNR@CNT displayed a high onset potential of 0.960 V and a half-wave potential of 0.819 V, which were only a little less than those of N-GNR@CNT (0.990 and 0.839 V) and even close to those of Pt/C (20 wt% Pt, 1.030 and 0.841 V). As shown in Supplementary Fig. 9, all these metal-free catalysts had a low H_2_O_2_ yield (<5.9%) and a high electron transfer number (*n* = 3.87–3.96), indicating a 4e^−^ ORR process in the alkaline electrolyte.

To further investigate the ORR mechanism, we carried out peroxide reduction reaction (PRR) tests for GNR@CNT, N-GNR@CNT (Fig. 3b), and Pt/C (Supplementary Fig. 10)^34^. It is clear that the N-GNR@CNT and Pt/C showed high PRR activity, whereas the GNR@CNT displayed very low PRR activity at H_2_O_2_ concentrations of 1.3 mM (the same concentration of saturated O_2_ in the electrolyte for the normal ORR test) and 10 mM. It suggests that the zigzag carbon did not prefer a “2e^−^ + 2e^−^” ORR process (O_2_ → H_2_O_2_ → H_2_O). Therefore, a direct 4e^−^ mechanism should dominate the ORR process of the GNR@CNT (zigzag carbon) in the alkaline electrolyte. The temperature dependence of catalytic activity was investigated for GNR@CNT and N-GNR@CNT in the range of 5–35 °C. As presented in Fig. 3c, the half-wave potential and the limiting current density of GNR@CNT increased with increasing operation temperature. In contrast, N-GNR@CNT showed an insensitivity to the temperature. Additionally, the GNR and N-GNR displayed the similar temperature dependence as GNR@CNT and N-GNR@CNT, respectively (Supplementary Fig. 11), further confirming the distinctively different active sites between GNR@CNT and N-GNR@CNT.

Our carbon catalysts were further characterized in an acid electrolyte, i.e. 0.5 M H_2_SO_4_. The capacitance tests in Supplementary Fig. 12 also confirmed the effects of ammonia treatment and CNT backbones, like in the alkline media. As shown in Fig. 3d, GNR@CNT exhibited a considerable activity with an onset potential of 0.760 V and a half-wave potential of 0.633 V although still lower than Pt/C (20 wt% Pt) in the acid electrolyte^35^. It is worth noting that both onset potential and limiting current of GNR@CNT were higher than those of N-GNR@CNT in acid, which was opposite to the tendency in alkaline media. This phenomenon could be explained by the protonation of pyridinic nitrogen of N-GNR@CNT in the acid solution. As previously reported, the pyridinic nitrogen could adsorb proton in acidic medium, which reduced the affinity of O_2_ to the active carbon atoms adjacent to the pyridinic N, thus decreasing the ORR activity of N-GNR@CNT^36,37^. In contrast, the GNR@CNT could be tolerant to the protonation reaction. The electron transfer number of GNR@CNT was 3.6–3.9, suggesting the formation of H_2_O_2_ byproduct (Supplementary Fig. 13). Different from the previous PRR results in the alkaline electrolyte (Fig. 3b), both GNR@CNT and N-GNR@CNT showed quite weak PRR currents (<0.4 mA cm^−2^) no matter with a high or low concentration of H_2_O_2_. This phenomenon indicates that GNR@CNT and N-GNR@CNT had no activity to PRR in the acid electrolyte, which differed from the Pt/C catalyst (Supplementary Fig. 14). Therefore, both GNR@CNT and N-GNR@CNT should undergo a direct 4e^−^ ORR with a negligible “2e^−^ + 2e^−^” process in acid. The temperature dependence of electrocatalytic activities for GNR@CNT, N-GNR@CNT, GNR, and N-GNR was illustrated in Fig. 3f and Supplementary Fig. 15-16. All these catalysts displayed increased ORR activities with increasing temperature from 5 to 35 °C. Since the practical PEMFCs are usually operated at tens of degrees Centigrade, this temperature dependence implied a promising future for metal-free electrocatalysts to achieve high PEMFC performance^38^.

**Fig. 4:**
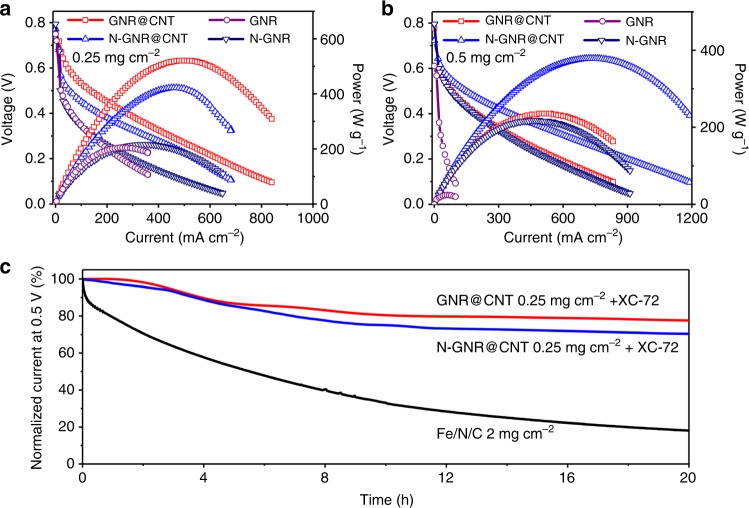
Proton exchange membrane fuel cell evaluation. Polarization and power density curves of graphene nanoribbons on carbon nanotubes (GNR@CNT), N-doped GNR@CNT (N-GNR@CNT), and N-doped graphene nanoribbons (N-GNR) as a function of the areal current density with cathode catalyst loading of **a** 0.25 mg cm^−2^ and **b** 0.50 mg cm^−2^ in a proton exchange membrane fuel cell (PEMFC); **c** stability of the indicated catalysts in PEMFC measured at 0.5 V. The absolute current densities before durability tests (at 100%) were 136, 80 and 1216 mA cm^−2^ for graphene nanoribbons on carbon nanotube (GNR@CNT), nitrogen-doped catalyst (N-GNR@CNT), and reference catalyst iron-nitrogen-carbon (Fe/N/C), respectively. Weight ratio of Nafion/catalyst/carbon black (XC-72) = 5/1/4. Cell: 80 °C; H_2_/O_2_: 80 °C, 100% relative humidity, 2 bar back pressure

After the half-cell characterization, GNR@CNT, GNR, N-GNR@CNT, and N-GNR were assembled into the membrane electrode assembly (MEA) for evaluation in an actual PEMFC. Per our experience, the well separation of GNR in the catalyst layer should be crucial for a good PEMFC performance. Here carbon black (XC-72) was added into the catalyst layer as a spacer to separate GNR. GNR@CNT exhibited the best polarization curve and the highest peak power density of 520 W g^−1^, better than 430 W g^−1^ of N-GNR@CNT at the catalyst loading of 0.25 mg cm^−2^ (Fig. 4a). N-GNR and GNR presented relatively poor polarization curves and low power performances (<200 W g^−1^) due to the lack of CNT backbones. To the best our knowledge, the obtained gravimetric peak power density of GNR@CNT in PEMFC is the highest value among all reported metal-free electrocatalysts and comparable to most NPMCs (Supplementary Table S4). Moreover, we found a strong dependence of fuel cell performance on catalyst loading. When the catalyst loading was doubled to 0.5 mg cm^−2^, the power density of GNR@CNT declined obviously (Fig. 4b), due to the worsened oxygen transport in the thick catalyst layer. For N-GNR@CNT, the performance decay was relatively small in comparison with GNR@CNT, which could be ascribed to the nanoholes on N-GNR@CNT (Fig. 2b, c) to facilitate the mass transport in the catalyst layer^39^. When expressed in the unit of W cm^−2^, GNR@CNT and N-GNR@CNT could reach 161 and 241 mW cm^−2^, respectively (Supplementary Fig. 17), which are among the top performance metal-free catalysts^16,40^. It should be noted that the open circuit voltage of nanoribbon catalysts as shown in Supplementary Table 5 were still less than that of Pt/C and most NPMCs. Finally, GNR@CNT, N-GNR@CNT, and a representative Fe/N/C catalyst were subject to a stability test in PEMFC at a constant voltage of 0.5 V with pure H_2_/O_2_ as fuel gases. As shown in Fig. 4c, N-GNR@CNT exhibited a more stable current performance than that of Fe/N/C, which was consistent with previous reports^16,41^. It is interesting to find that the GNR@CNT possessed an even better PEMFC stability than N-GNR@CNT at 0.5 V. To the best of our knowledge, this is the only zigzag carbon that exhbits both high activity and stability in a PEMFC.

The observed high PEMFC performance prompted us to identify the active sites of GNR@CNT. Since both the defective carbon atoms and the oxidized defect carbon atoms are active to ORR^19,42^ and defective carbon atoms could be oxidized after long exposure to air, we prepared a couple of samples with controlled oxidative defects as active sites. GNR@CNT was protected under Ar atmosphere during the synthesis, storage, and ink preparation, while the control sample was exposed to air for 5 days (denoted as GNR@CNT-5days) after the synthesis to enhance the oxidation^43^. It has been previously reported that the enhanced oxidation on CNT could decrease the *sp*^2^ peak and increase the *sp*^3^ peak in the C 1*s* XPS spectrum, accompanied by a hydroxyl group (C–OH) transferring to a carboxyl group (O = C–O) and then to CO_2_ and H_2_O^44,45^. The decreased *sp*^2^/*sp*^3^ ratio and increased O = C–O/H–O–H groups indicated a successful oxidation of GNR@CNT-5days, as shown in Supplementary Fig. 18 and Table 1. In the acid electrolyte, the ORR activity of GNR@CNT-5days was obviously lower than that of GNR@CNT in terms of half-wave potential, current density, electron transfer number, and H_2_O_2_ yield, as shown in Supplementary Fig. 19. Hence, the observed high ORR activity of GNR@CNT in PEMFC should be mainly ascribed to the zigzag carbon rather than the oxidized zigzag carbon.

### Theoretical calculations of free energy

**Fig. 5:**
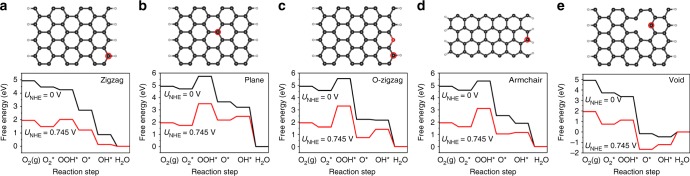
Theoretical calculations. Models (top) and the corresponding free energy diagrams (bottom) for cycled carbon atoms at electrode potential *U*_NHE_ = 0 and 0.745 V in 0.5 M H_2_SO_4_ (NHE normal hydrogen electrode, RHE reversible hydrogen electrode, *U*_NHE_ = *U*_RHE_ − 0.0591 × pH, pH = 0.25). **a** Carbon atom at zigzag edge, **b** carbon atom in basal plane, **c** carbon atom at O-doped zigzag edge, **d** carbon atom at armchair edge, and **e** carbon atom near a void

To further identify the active site on GNR in the acid electrolyte, a DFT method was employed to calculate the free energy of ORR process on the representative carbon atoms. Although zigzag edges of GNR@CNT could be decorated by a small amount of –O (or –OH), the OH–C could be excluded since it could not adsorb O_2_ for ORR^42^. So, five types of carbon atoms on GNR were deduced as possible active sites, including (a) zigzag carbon atom, (b) carbon atom in basal plane, (c) carbon atom near O-doped zigzag edge, (d) carbon atom at armchair edge, and (e) carbon atom near a void as shown in Fig. 5. The minimum energy pathways for ORR on each type of carbon atoms were calculated (for more computational details see Supporting Information). As shown in Fig. 5a, the most active site under pH = 0.25 (0.5 M H_2_SO_4_) was figured out to be the zigzag carbon atom (a) (see Supplementary Fig. 20 for ORR process on zigzag carbon), which possessed the smallest limiting ΔG_OOH*_ of 0.54 eV for O_2_* to OOH* at *U*_NHE_ = 0.745 V (onset potential for GNR@CNT in 0.5 M H_2_SO_4_, * stands for an active site on the catalyst). The obtained ΔG_OOH*_ is very close to the reported value for pure graphene in 0.1 M KOH^46^. In contrast, the carbon atom in GNR basal plane, the carbon atom near O-doped zigzag edge, and the armchair carbon had significantly enlarged free energy for the step of O_2_* to OOH*, and the corresponding Δ*G* were 1.78, 1.71, and 1.46 eV, respectively (Fig. 5b–d). As to the carbon atom near a void, the rate-limiting Δ*G* was 1.24 eV for the step of OH* to H_2_O. These large Δ*G* hindered the formation of OOH* (the key intermediate) or H_2_O, and thus excluded their possibility as ORR active sites at the onset potential.

## Discussion

In summary, the zigzag-edged GNRs with CNT backbones (GNR@CNT) have been developed by partially unzipping MWCNTs and used as the metal-free ORR electrocatalyst for the H_2_/O_2_ PEMFCs. With the assistance of a CNT backbone and carbon black spacer, the mass transport in the catalyst layer was enhanced, coupled with an enhanced exposure of the zigzag carbon active sites along the GNR edge. Among all carbon-based metal-free electrocatalysts, GNR@CNT achieved the highest gravimetric power density of 520 W g^−1^ in PEMFCs to the best of our knowledge. More importantly, GNR@CNT demonstrated an improved stability compared to a Fe/N/C catalyst in PEMFCs. DFT calculations coupled with experimental results revealed that zigzag carbon atoms possessed the higher electrocatalytic activity to ORR, than oxidized zigzag carbon, basal plane carbon, armchair edge carbon, and carbon atom near a void in acid electrolyte. This study demonstrates a great potential for defective graphitic carbon to be used in PEMFC application.

## Methods

### Reagents

MWCNTs (length: 0.5–2 μm, outer diameter: 10–20 nm) were obtained from Chengdu Organic Chemicals Co. Ltd. (Chengdu). Concentrated sulfuric acid, concentrated hydrochloric acid, potassium permanganate, and hydrogen peroxide were obtained from Sinopharm Chemical Reagent Beijing Co. Ltd. (Beijing). Pt/C (20 wt% Pt) and Pt/C (40 wt% Pt) were purchased from Sigma-Aldrich. Besides CNTs, all of the other materials were of analytical grade and used without further purification.

### Preparation of catalysts

To purify nanotubes (to remove metal impurities), raw MWCNT was stirred in concentrated HCl for 24 h. A unit of 200 mg purified MWCNT was put into a flask and mixed with 36 mL concentrated H_2_SO_4_. After 30 min of ultra-sonication and 1 h stirring, 800 mg KMnO_4_ (1600 mg for completed unzipping) was slowly added into the mixture and stirred for 1 h. The mixture was further heated in oil bath at 55 °C for 15 min and then at 70 °C for 45 min. After the mixture cooled down to the room temperature, it was poured into 100 mL ice water (containing 3 mL 30 wt% H_2_O_2_) and then washed in 10 wt% HCl solution five times and collected by centrifuge at 12 000 rpm, and finally went through a dialysis for 1 week. Sample solution was freeze-dried and annealed in Ar atmosphere at 900 °C for 30 min for defective carbon GNR@CNT and GNR, or in NH_3_ atmosphere at 800 °C for 30 min for N-doped catalysts N-GNR@CNT and N-GNR.

Fe/N/C was synthesized according to literature^47^. Specifically, we performed ball milling on 100 mg zeolitic imidazolate framework (ZIF-8) together with 10 mg tris(1, 10-phenanthroline) iron(II) perchlorate ion for 1 h, which was subsequently heated in Ar at 1000 °C for 1 h, and then at 900 °C under NH_3_ for 15 min.

### Electrochemical measurements in half cell

Electrochemical properties were characterized using rotating ring disc electrode (model 3A, ALS Co.) in a three-electrode beaker cell equipped with a Pt wire counter electrode and a saturated Ag/AgCl reference electrode. Here 0.1 M KOH or 0.5 M H_2_SO_4_ was used as the electrolyte. Before the electrochemical experiments, the Ag/AgCl reference electrode was calibrated and potentials were converted to reversible hydrogen electrode (RHE) scale (*V*_RHE_ = *V*_Ag/AgCl_ + 0.0591 × pH + 0.197 V). The catalyst ink was prepared by dispersing 1 mg catalyst in Nafion solution (100 μL, 1 mg mL^−1^). The working electrode was prepared by dropping the catalyst ink (5 μL) onto the glassy carbon disk electrode (4 mm diameter) and then drying at room temperature. Loading amount of the catalysts was 398 μg cm^−2^ (Pt/C (20 wt% Pt) 99 μg cm^−2^). The LSV were recorded at a scan rate of 10 mV s^−1^ in an O_2_-saturated electrolyte (rotating speed: 1600 rpm, scan range: 0.2–1.1 *V*_RHE_). The PRR polarization curves were recorded at 10 mV s^–1^ and 1600 rpm rotation speeds in Ar-saturated 0.1 M KOH and 0.5 M H_2_SO_4_ solution with 1.3 and 10 mM H_2_O_2_ electrolytes. ORR and PRR results were presented after the subtraction of the capacitive background measured in Ar-saturated 0.1 M KOH or 0.5 M H_2_SO_4_ electrolyte.

The electron transfer number (*n*) and OOH^−^ intermediate production percentage (%OOH^−^) were determined by:

1 $${n} = 4 \times \frac{{I_{\mathrm{d}}}}{{I_{\mathrm{d}} + I_{\mathrm{r}}/N}}$$

2 $${\mathrm{\% OOH}}^ - = 200 \times \frac{{I_{\mathrm{r}}/N}}{{I_{\mathrm{d}} + I_{\mathrm{r}}/N}}$$

where *I*_d_ is the disk current, *I*_r_ is the ring current, and *N* is the current collection efficiency of the Pt ring, which was determined to be 0.4.

### PEMFC test

The synthesized catalyst was used as the cathode for a PEMFC. A typical catalyst ink was prepared by dispersing 1.25 mg catalyst, 5 mg XC-72, and 125 mg Nafion solution (5 wt% Nafion) in deionized water (300 μL) and isopropanol (600 μL) by sonication and stirring. The catalyst ink was coated on 5 cm^2^ carbon paper at a loading of 0.25 mg cm^−2^. For the anode, the catalyst was a commercial Pt/C (40 wt% Pt) catalyst with a loading of 0.2 mg Pt per cm^2^. MEAs were fabricated by hot-pressing the anode, cathode, and a Nafion membrane (model: NRE 211) at 1.5 MPa for 120 s at 130 °C. The performance of the fuel cell was assessed using a Model 850e fuel cell test system (Scribner Associates Inc.) operated at 80 °C. The H_2_ and O_2_ flow rates were 0.3 and 0.5 L min^−1^ at 100% relative humidity and 2 bar back pressure.

### Computational methods

The DFT calculation was performed within a generalized gradient approximation designed by Perdew-Burke-Ernzerhof, as implemented in the Vienna ab initio simulation package. The projector-augmented wave method is used to describe the ionic potentials. The unit box was built for calculations with a volume of 9.86 Å × 29.00 Å × 18.00 Å for zigzag carbon, basal plane carbon, oxidized zigzag carbon, and carbon near a void, or 8.54 Å × 29.00 Å × 18.00 Å for armchair carbon. The plane wave basis set has a high cutoff energy of 500 eV throughout the computation. The *K*-point sampling of the Brillouin zone was obtained using a 1 × 1 × 1 grid generating meshes with their origin centered at the gamma (Γ) point. All calculations were spin-polarized and the force convergence criterion for atomic relaxation was 0.01 eV Å^−1^. The ORR can proceed either through a two-step two-electron pathway that reduces O_2_ to H_2_O_2_ or via a direct four-electron process in which O_2_ is directly reduced to H_2_O without involvement of an H_2_O_2_ intermediate. Here we study the complete reduction cycle because GNR@CNT shows a transfer number of about 3.7 in ORR, which is close to the four-electron process. In the acidic environment, the ORR can be written as:

3 $${\mathrm{O}}_2\left( {\mathrm{g}} \right) + ^ \ast \to {\mathrm{O}}_2^ \ast$$

4 $${\mathrm{O}}_{\mathrm{2}}^ \ast {\mathrm{ + H}}^{\mathrm{ + }}{\mathrm{ + e}}^{\mathrm{ - }} \to {\mathrm{OOH}}^ \ast$$

5 $${\mathrm{OOH}}^ \ast + {\mathrm{H}}^ + + {\mathrm{e}}^ - \to {\mathrm{O}}^ \ast + {\mathrm{H}}_{\mathrm{2}}{\mathrm{O }}\left( 1 \right)$$

6 $${\mathrm{O}}^ \ast {\mathrm{ + H}}^{\mathrm{ + }}{\mathrm{ + e}}^{\mathrm{ - }} \to {\mathrm{OH}}^ \ast$$

7 $${\mathrm{OH}}^ \ast {\mathrm{ + H}}^{\mathrm{ + }}{\mathrm{ + e}}^{\mathrm{ - }} \to {\mathrm{H}}_{\mathrm{2}}{\mathrm{O }}\left( {\mathrm{l}} \right){\mathrm{ + }}^ \ast$$

where * stands for an active site on the graphene surface or at zigzag edge, (l) and (g) refer to liquid and gas phases, respectively, and O*, OH*, and OOH* are adsorbed intermediates.

It was reported that the ORR rate-determining step can either be the O_2_* to OOH* (Eq. 4) or OH* to water (Eq. 7)^48^. The overpotential of the ORR processes can be determined by examining the reaction free energies (Δ*G*) of the different elementary steps. For each step the reaction free energy is defined as the difference between free energies of the initial and final states and is given by the expression:

8 $${{\Delta G = \Delta E + \Delta {\mathrm{ZPE}}-T\Delta S + \Delta G}}_{\mathrm{U}}{{ + \Delta G}}_{{\mathrm{pH}}}$$

Where Δ*E* is the reaction energy of reactant and product molecules adsorbed on catalyst surface obtained from DFT calculations. Δ*G*_U_ = −*eU*, where *U* is the potential at the electrode. *T* is the temperature and *e* is the transferred charge. ΔZPE is the change of zero-point energy computed by density functional perturbation theory. Δ*S* is the entropy change after the adsorption from the DFT simulation. Δ*G*_pH_ (here pH is 0.25) is the correction of the H^+^ free energy calculated by the concentration dependence of the entropy, in which *k*_B_ is the Boltzmann constant.

9 $${{\Delta G}}_{{\mathrm{pH}}}{{ = - k}}_{\mathrm{B}}{{T{\mathrm{ln}}}}\left[ {{\mathrm{H}}^ + } \right]$$

### Physical characterization

The morphology and structure were characterized by TEM (JEM-2100F, Japan). XPS was performed on an ESCALAB 250Xi (Thermo Fisher Scientific Company, US) using a monochromated Al Kα source and a pass energy of 50 eV at a base pressure of 1 × 10^−8^ mbar. FT-IR spectra were recorded using a Nicolet iN10 MX (Thermo Fisher Scientific Company).

## Electronic supplementary material

Supplementary Information

## Data Availability

The data that support the findings of this study are available from the corresponding author upon reasonable request.
