# Supplementary material for: Zigzag carbon as efficient and stable oxygen reduction electrocatalyst for proton exchange membrane fuel cells
Source: Nat Commun. 2018 Sep 19;9:3819. doi: 10.1038/s41467-018-06279-x (PMC6145918; doi:10.1038/s41467-018-06279-x)
Supplement: Supplementary file 1 — Supplementary Information [file 41467_2018_6279_MOESM1_ESM.pdf]

# Supplementary Information

## Zigzag Carbon as Highly Efficient and Stable Electrocatalyst for Oxygen Reduction Reaction in PEMFC

**Xue et al.**

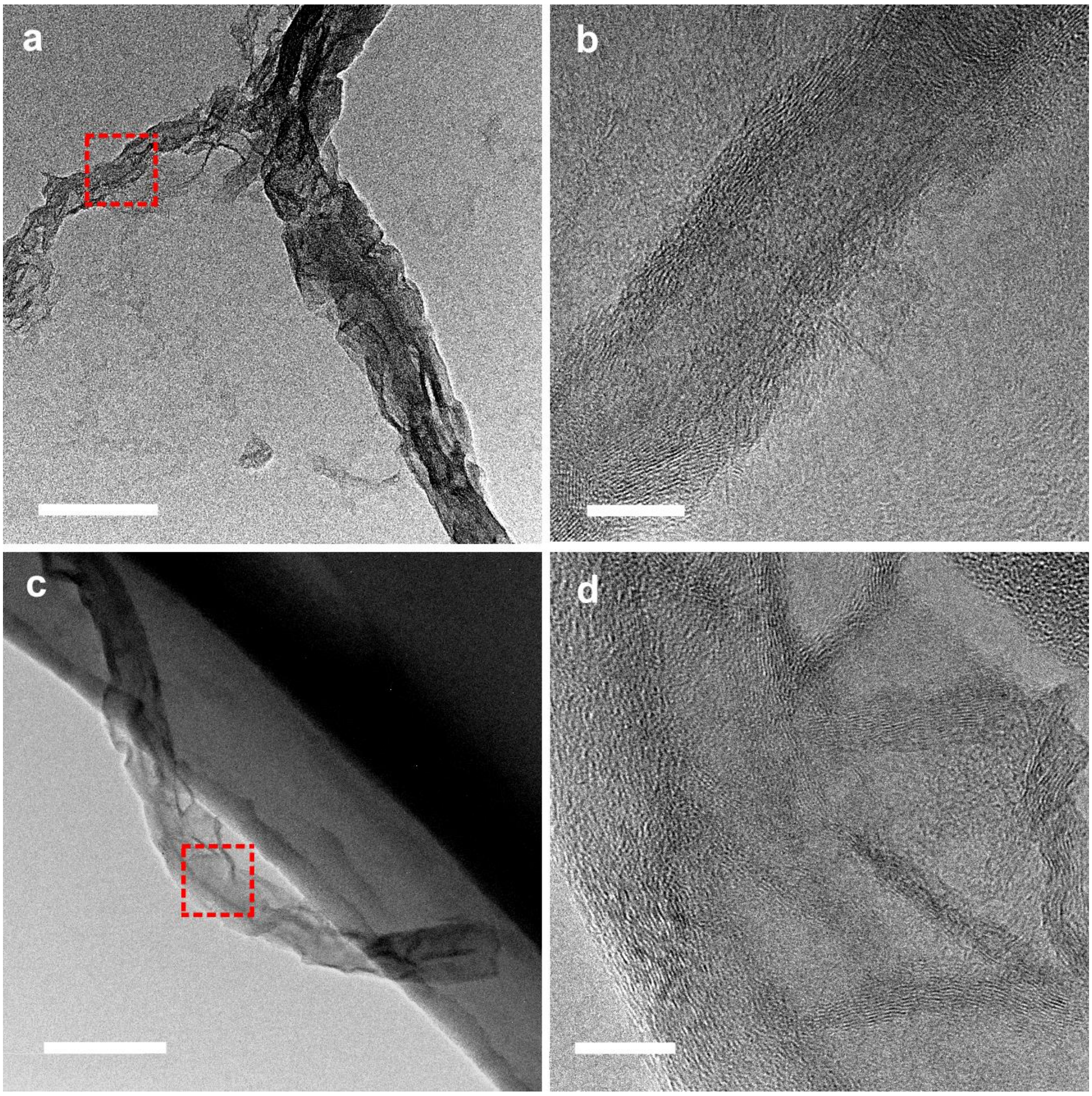


**Supplementary Figure 1 | Morphologies of oxidized-GNR@CNT and oxidized-GNR before annealing.** TEM images of (a-b) partially unzipped oxidized-GNR@CNT, and (c-d) fully unzipped oxidized-GNR. Scale bar in a, c is 100 nm, and in b, d is 10 nm.

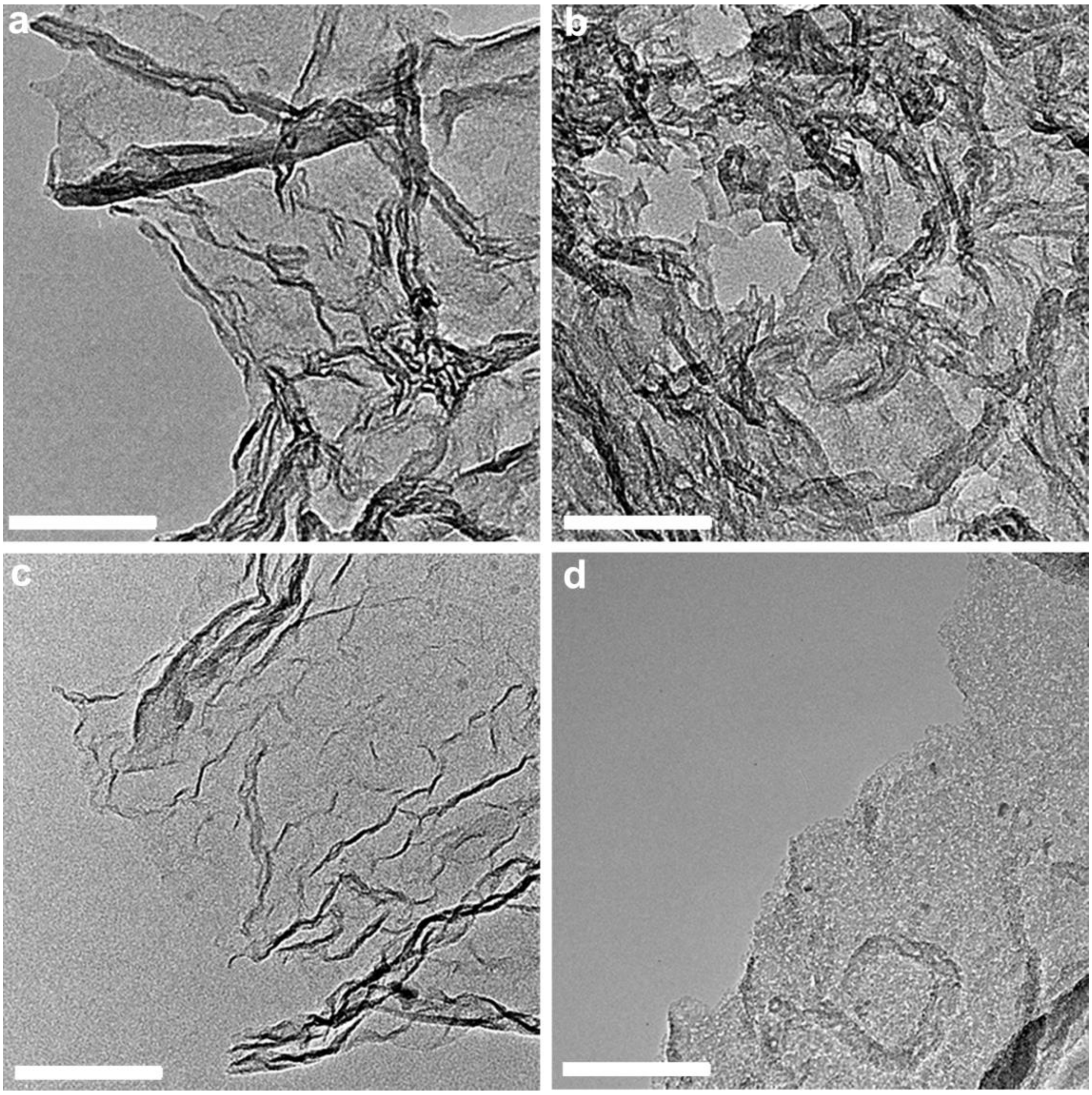


**Supplementary Figure 2 | Morphologies of four samples after annealing.** TEM images of (a) GNR@CNT, (b) N-GNR@CNT, (c) GNR and (d) N-GNR. Scale bar: 100 nm.

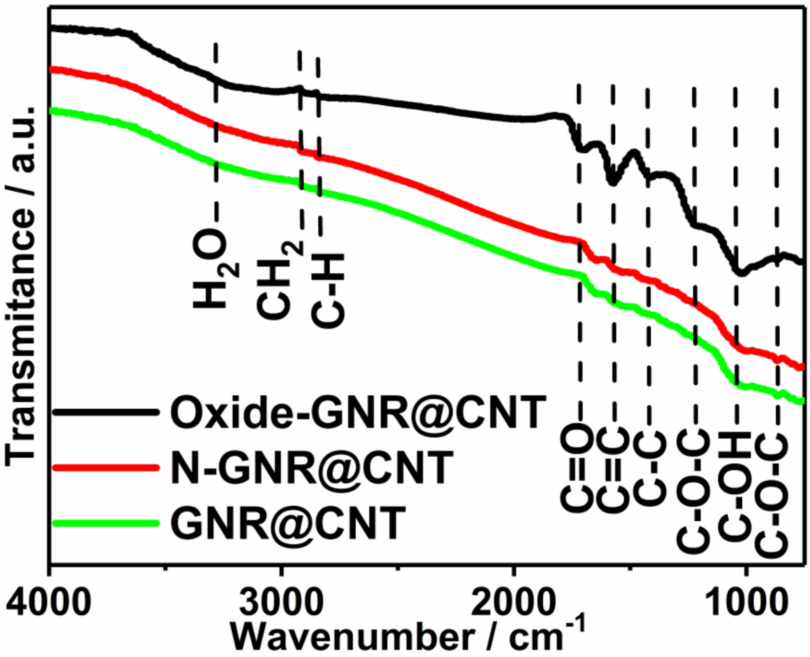


**Supplementary Figure 3 | FT-IR spectra of Oxide-GNR@CNT, GNR@CNT and N-GNR@CNT.**

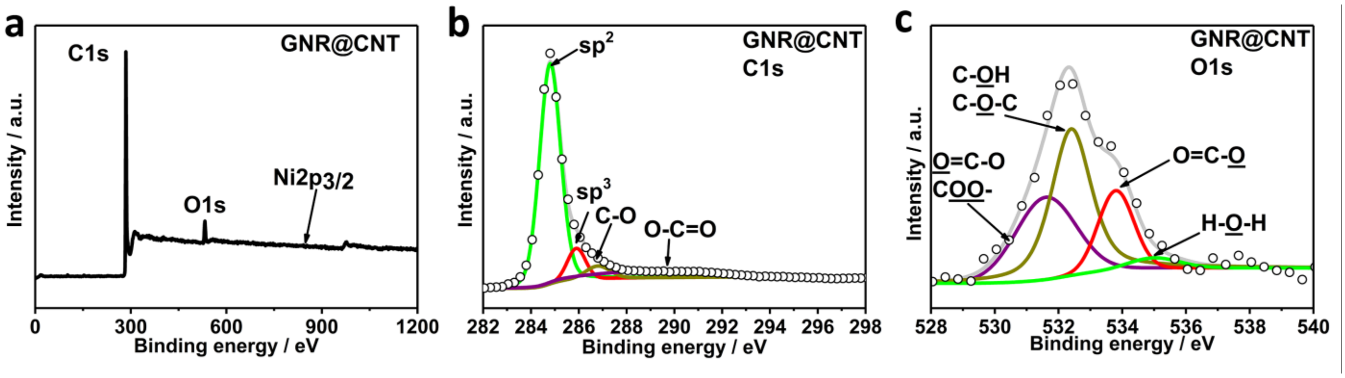


**Supplementary Figure 4 | XPS spectra of GNR@CNT.** (a) survey, and fine spectra of (b) C1s and (c) O1s.

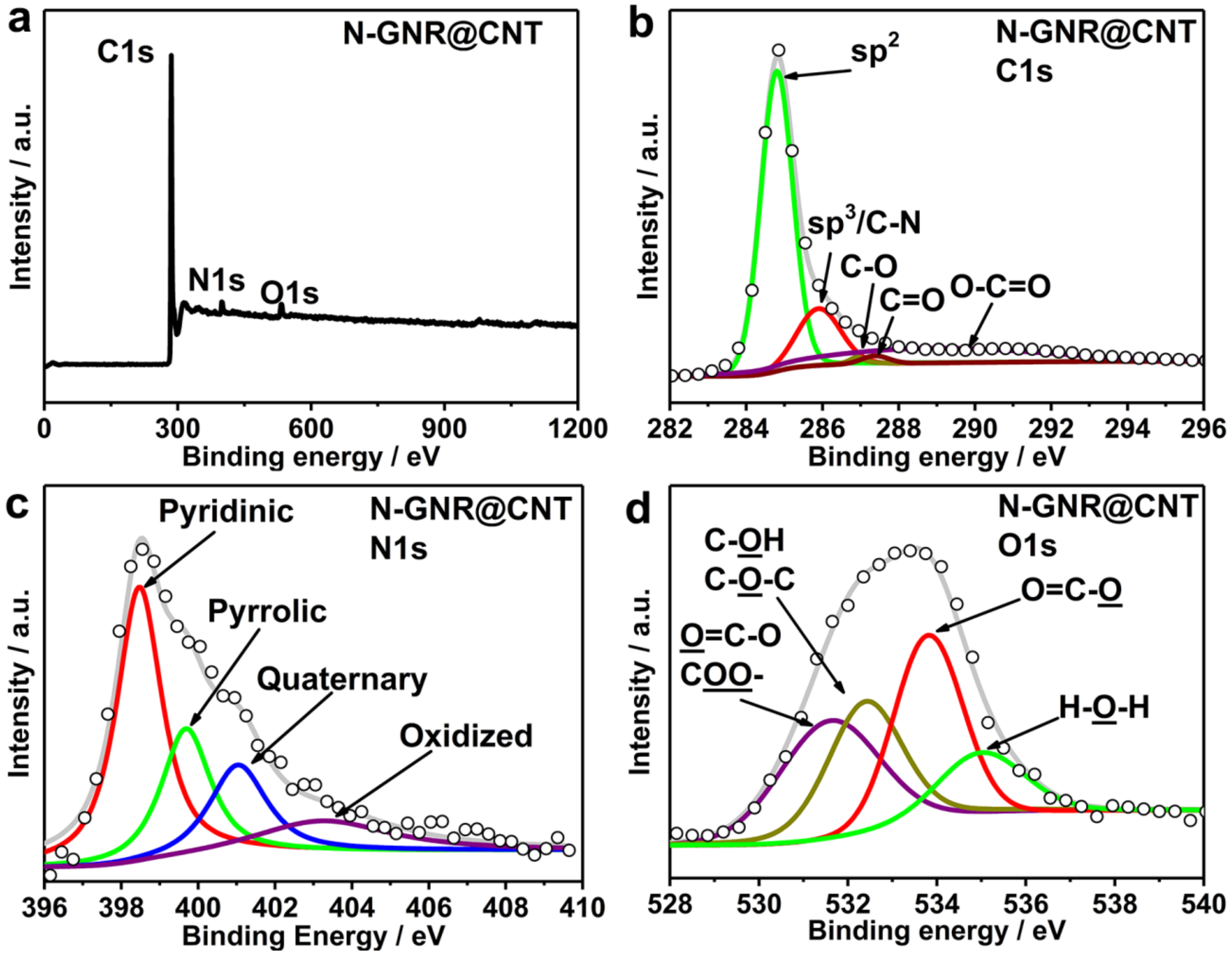


**Supplementary Figure 5 | XPS spectra of N-GNR@CNT.** (a) survey, and fine spectra of (b) C1s, (c) N1s and (d) O1s.

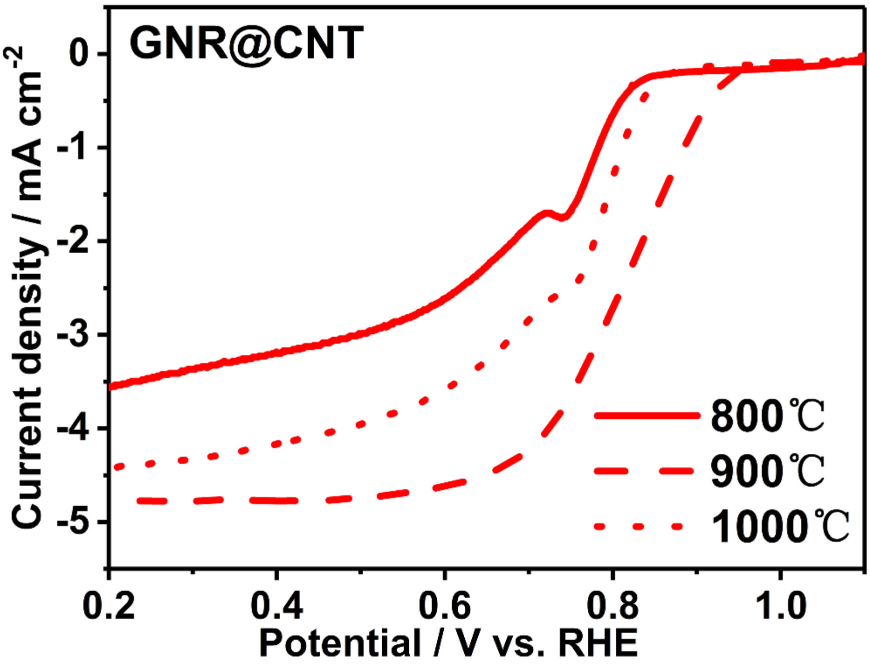


**Supplementary Figure 6 | LSV curves of GNR@CNT annealed at different temperatures in Ar.** The ORR were measured in $O_2$-saturated 0.1 M KOH at 1600 rpm. Scan rate: 10 mV $s^{-1}$.

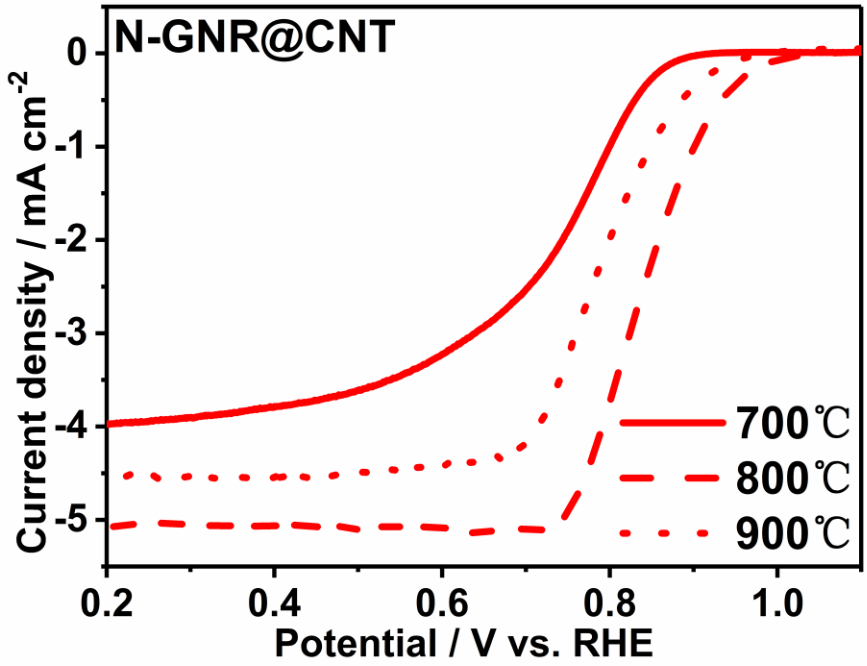


**Supplementary Figure 7 | LSV curves of N-GNR@CNT annealed at different temperatures in $NH_3$.**

The ORR were measured in $O_2$-saturated 0.1 M KOH at 1600 rpm. Scan rate: 10 mV $s^{-1}$.

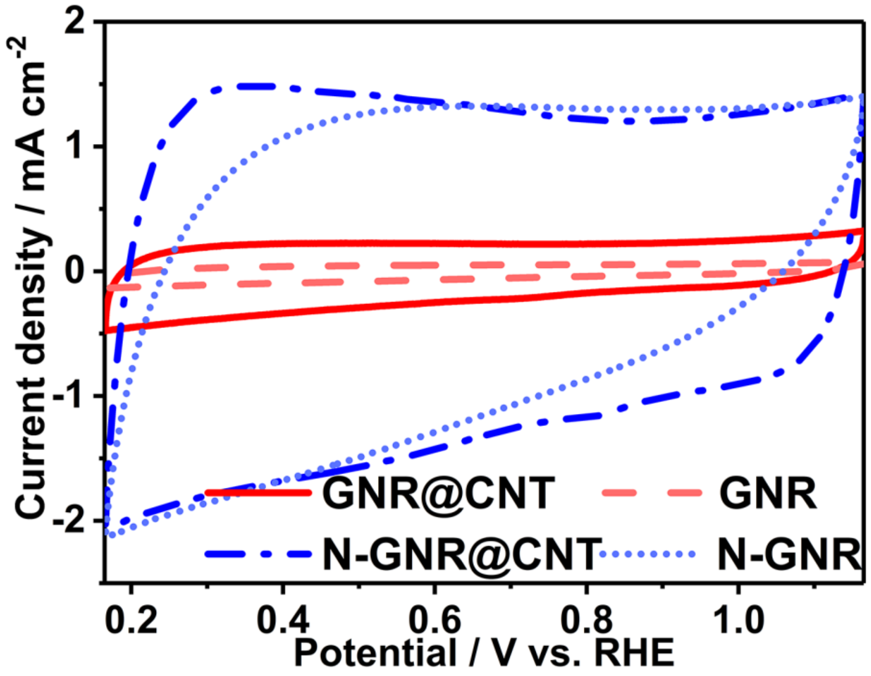


**Supplementary Figure 8 | CV curves of four samples measured in $N_2$-saturated 0.1 M KOH.** Scan rate: 50 mV $s^{-1}$.

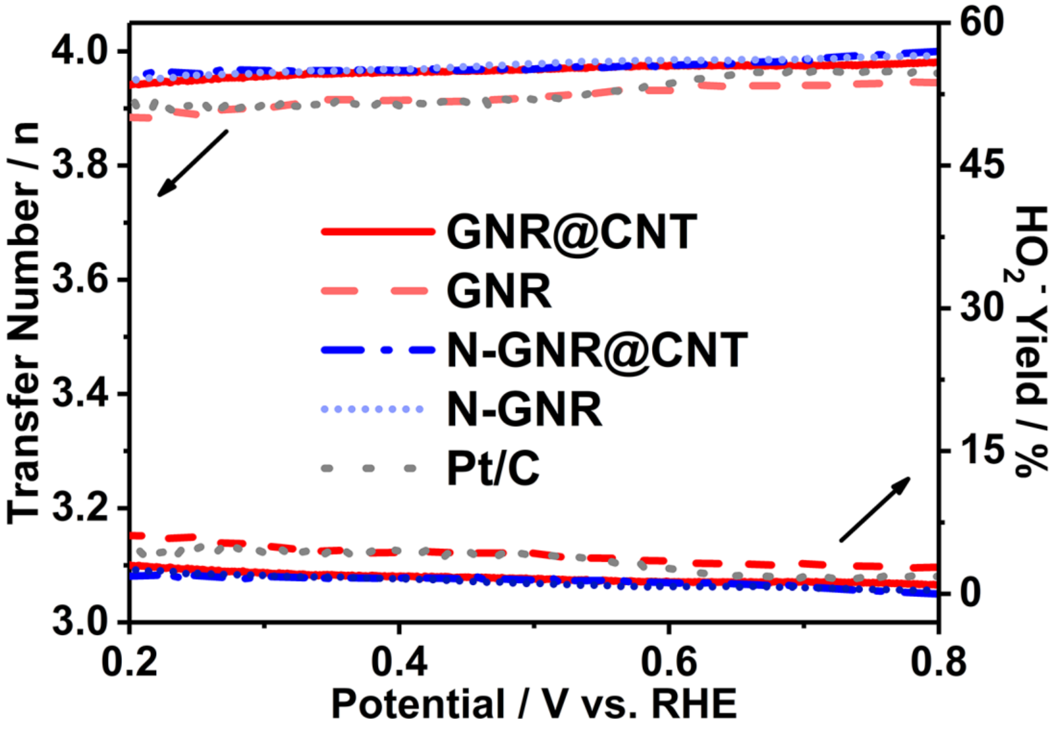


**Supplementary Figure 9 | Electron transfer number and $HO_2^-$ yield of GNR@CNT, GNR, N-GNR@CNT, N-GNR and Pt/C in $O_2$-saturated 0.1 M KOH at 1600 rpm.** Scan rate: 10 mV $s^{-1}$.

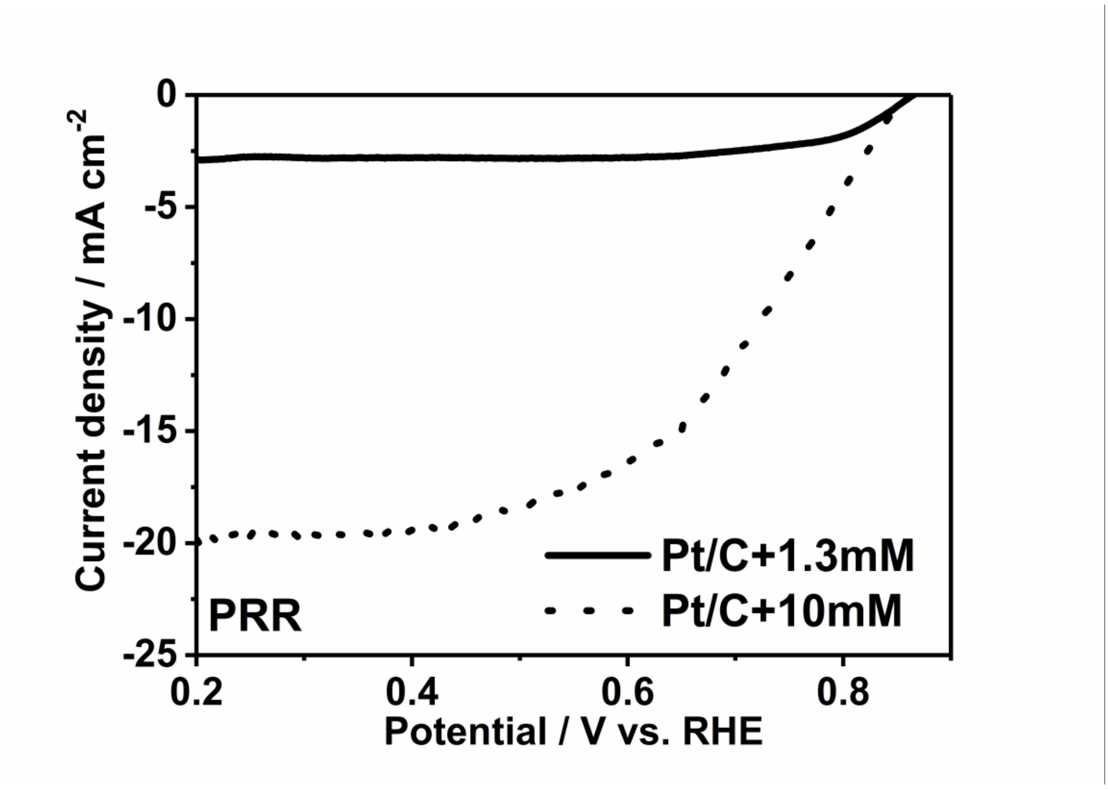


**Supplementary Figure 10 | PRR polarization curves of Pt/C(20wt% Pt) measured in Ar-saturated 0.1 M KOH at 1600rpm.** Scan rate: 10 mV $s^{-1}$.

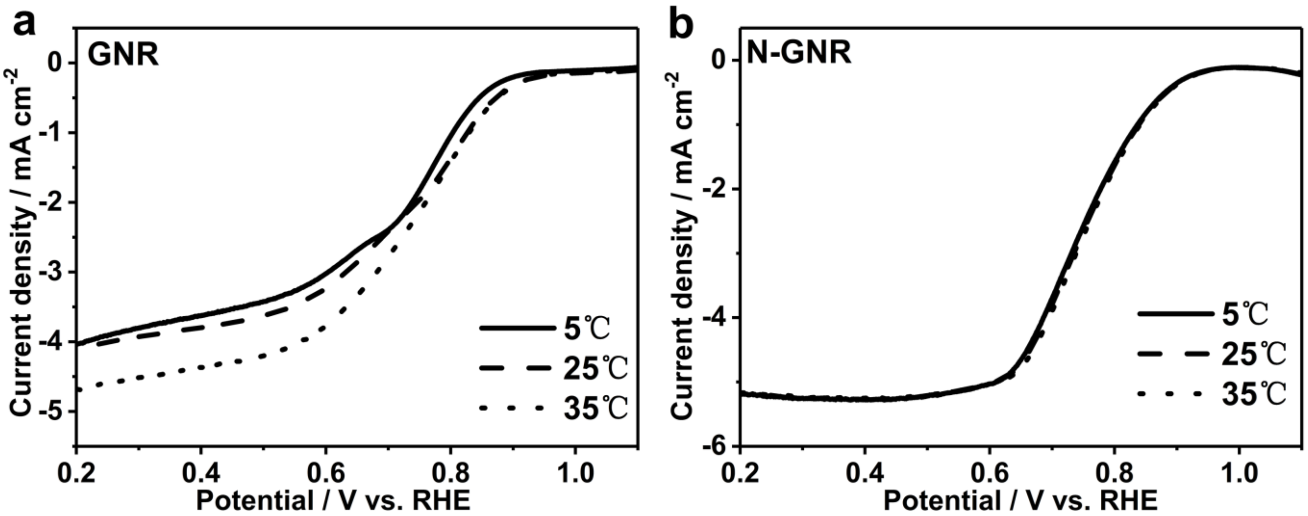


**Supplementary Figure 11 | ORR responses to the testing temperature in alkaline.** LSV curves of a) GNR and b) N-GNR measured at different temperatures in $O_2$-saturated 0.1 M KOH at 1600 rpm. Scan rate: 10 mV $s^{-1}$.

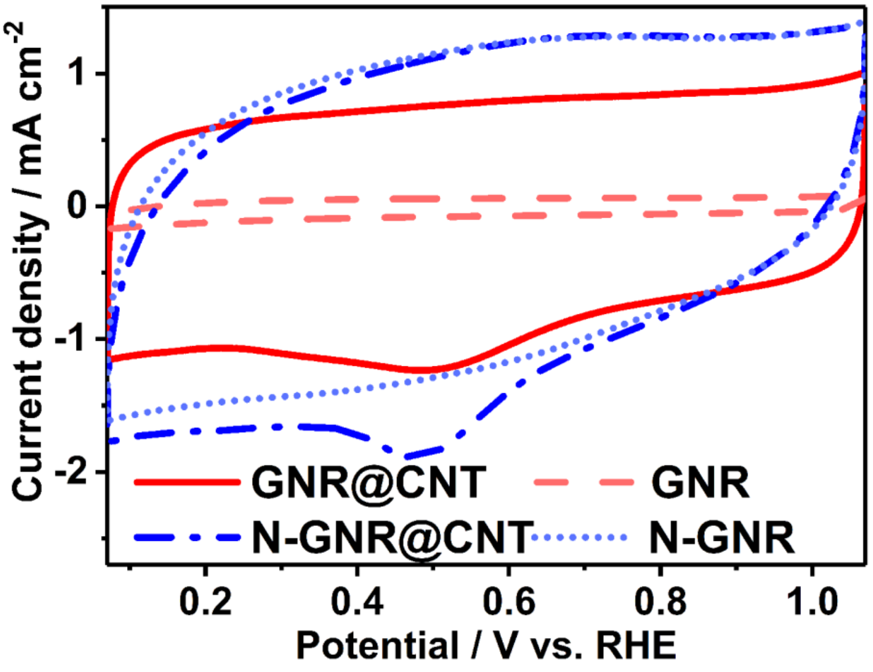


**Supplementary Figure 12 | CV curves of indicated samples measured in $N_2$-saturated 0.5 M $H_2SO_4$.** Scan rate: 50 mV $s^{-1}$.

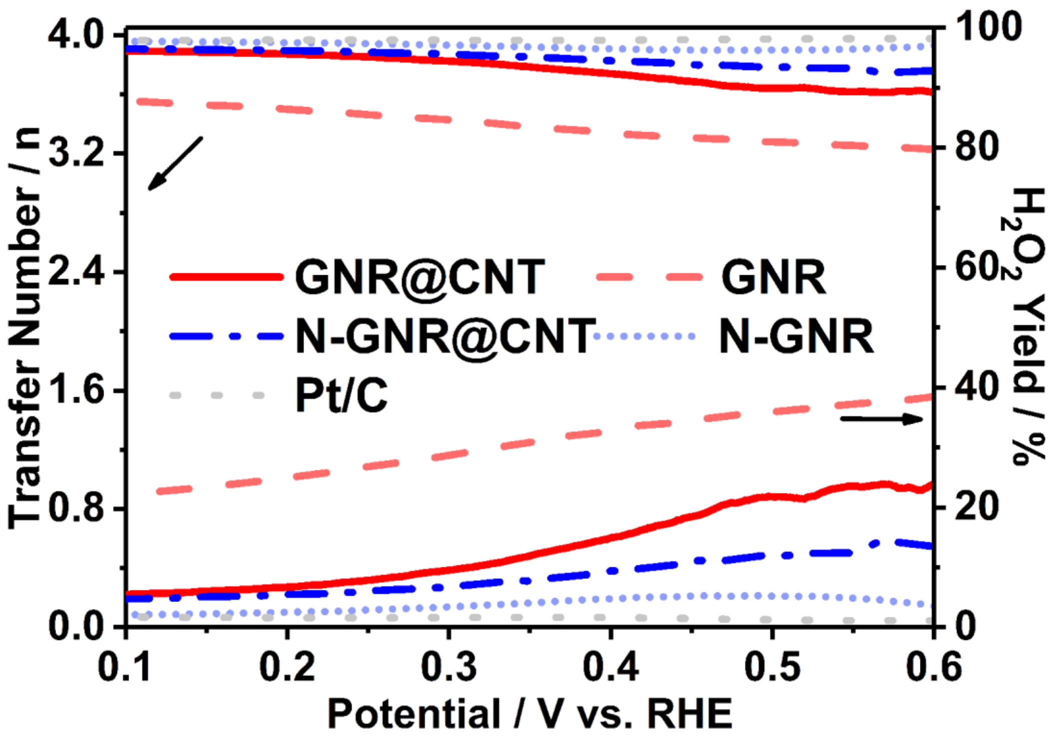


**Supplementary Figure 13 | Electron transfer number and $H_2O_2$ yield of GNR@CNT, GNR, N-GNR@CNT, N-GNR and Pt/C in $O_2$-saturated 0.5 M $H_2SO_4$ at 1600 rpm.** Scan rate: 10 mV $s^{-1}$.

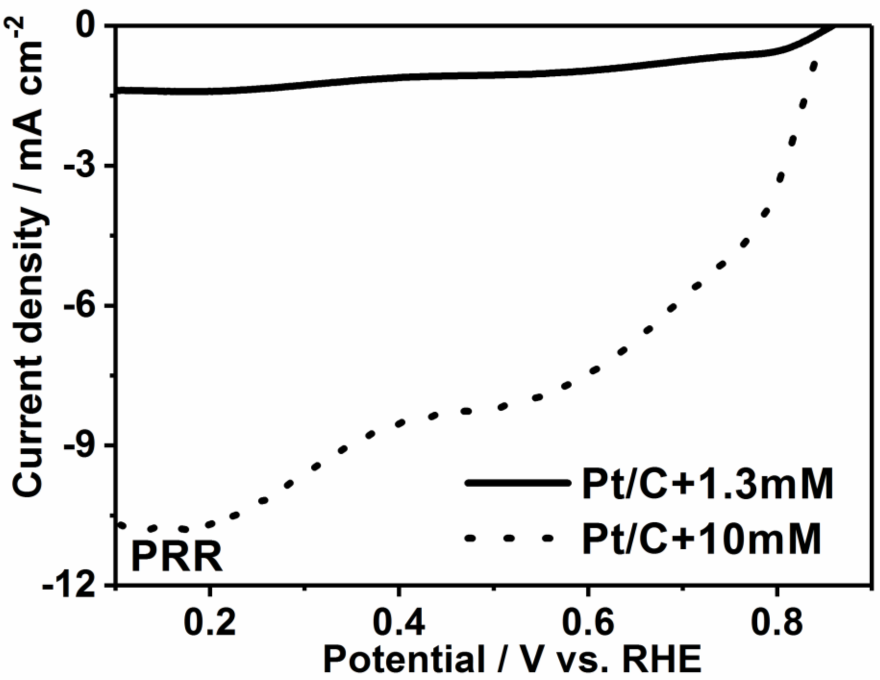


**Supplementary Figure 14 | PRR polarization curves of Pt/C(20wt% Pt) measured in 0.5 M $H_2SO_4$ at 1600 rpm.** Scan rate: 10 mV $s^{-1}$.

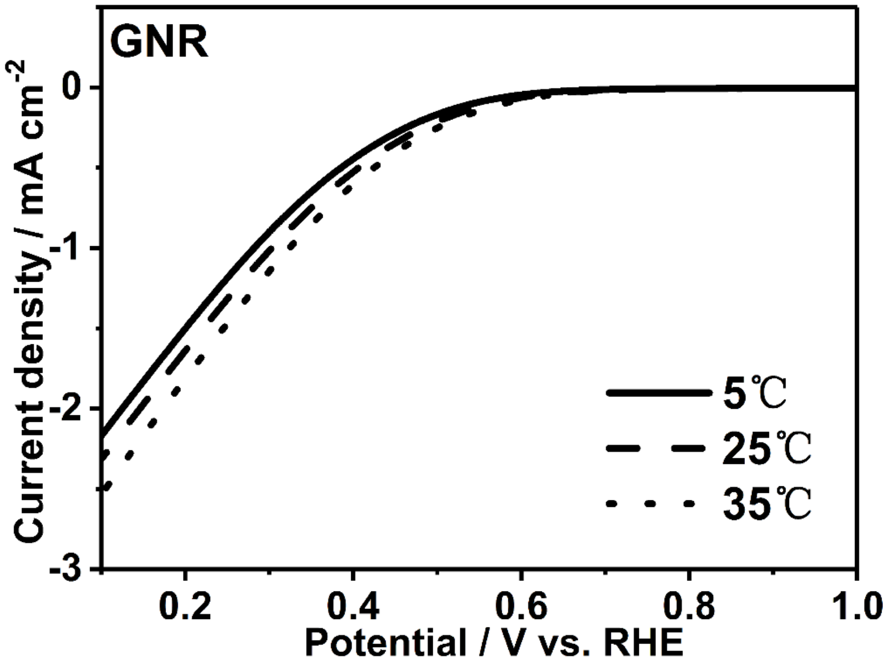


**Supplementary Figure 15 | LSV curves of GNR measured at different temperatures in $O_2$-saturated 0.5 M $H_2SO_4$ at 1600 rpm.** Scan rate: 10 mV $s^{-1}$.

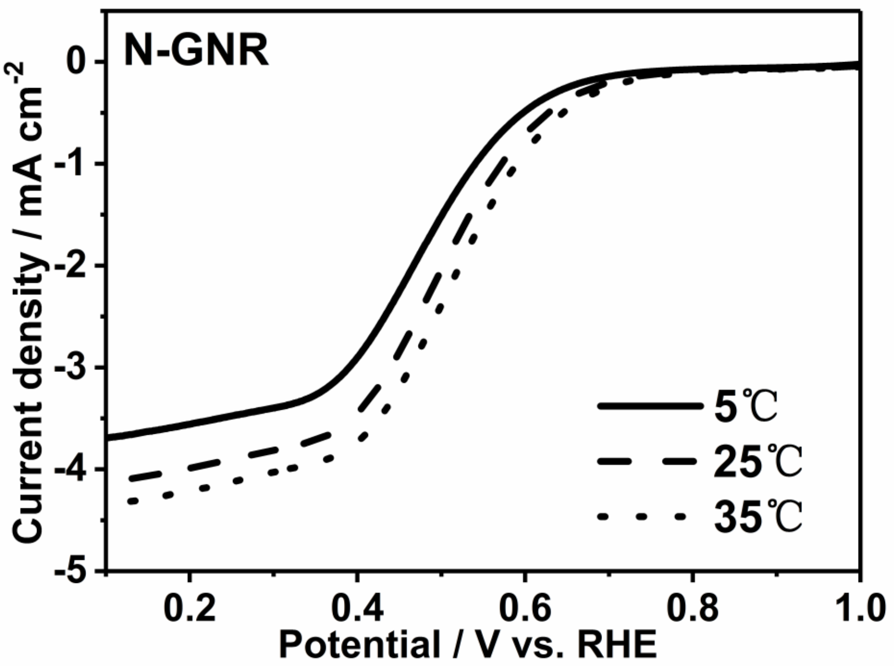


**Supplementary Figure 16 | LSV curves of N-GNR measured at different temperatures in $O_2$-saturated 0.5 M $H_2SO_4$ at 1600 rpm.** Scan rate: 10 mV $s^{-1}$.

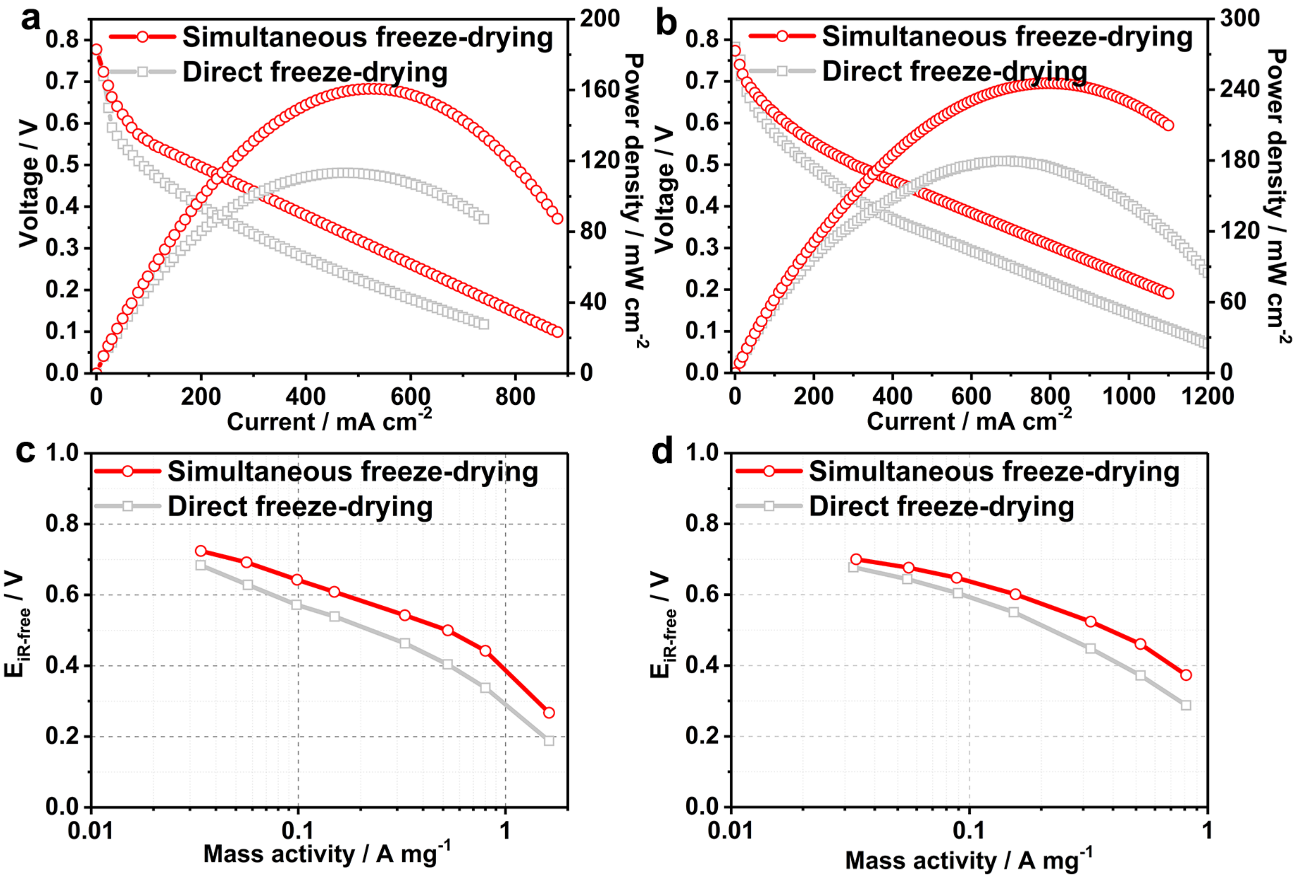


**Supplementary Figure 17 | Polarization and power density curves of** a) GNR@CNT+XC-72 (0.4+1.6 mg cm$^{-2}$) and b) N-GNR@CNT+XC-72 (0.9+2.2 mg cm$^{-2}$), and polarization curves expressed by $E_{iR\text{-}free}$ vs Log mass activity of c) GNR@CNT+XC-72 (0.4+1.6 mg cm$^{-2}$) and d) N-GNR@CNT+XC-72 (0.9+2.2 mg cm$^{-2}$) as cathode in PEMFC measured with 2 bar $H_2$–$O_2$, 100% RH, 80 °C.

Simultaneous freeze-drying: oxidized-GNR@CNT was mixed with XC-72 (spacer) first and then the mixture was freeze-dried and pyrolized. Direct freeze-drying: oxidized-GNR@CNT was freeze-dried and pyrolized to GNR@CNT or N-GNR@CNT first, and then they were mixed with XC-72 for catalyst ink. The PEMFC performance could be improved by enhancing the mass transfer of catalyst layer, through freeze-drying the catalyst precursor together with XC-72 before pyrolysis.

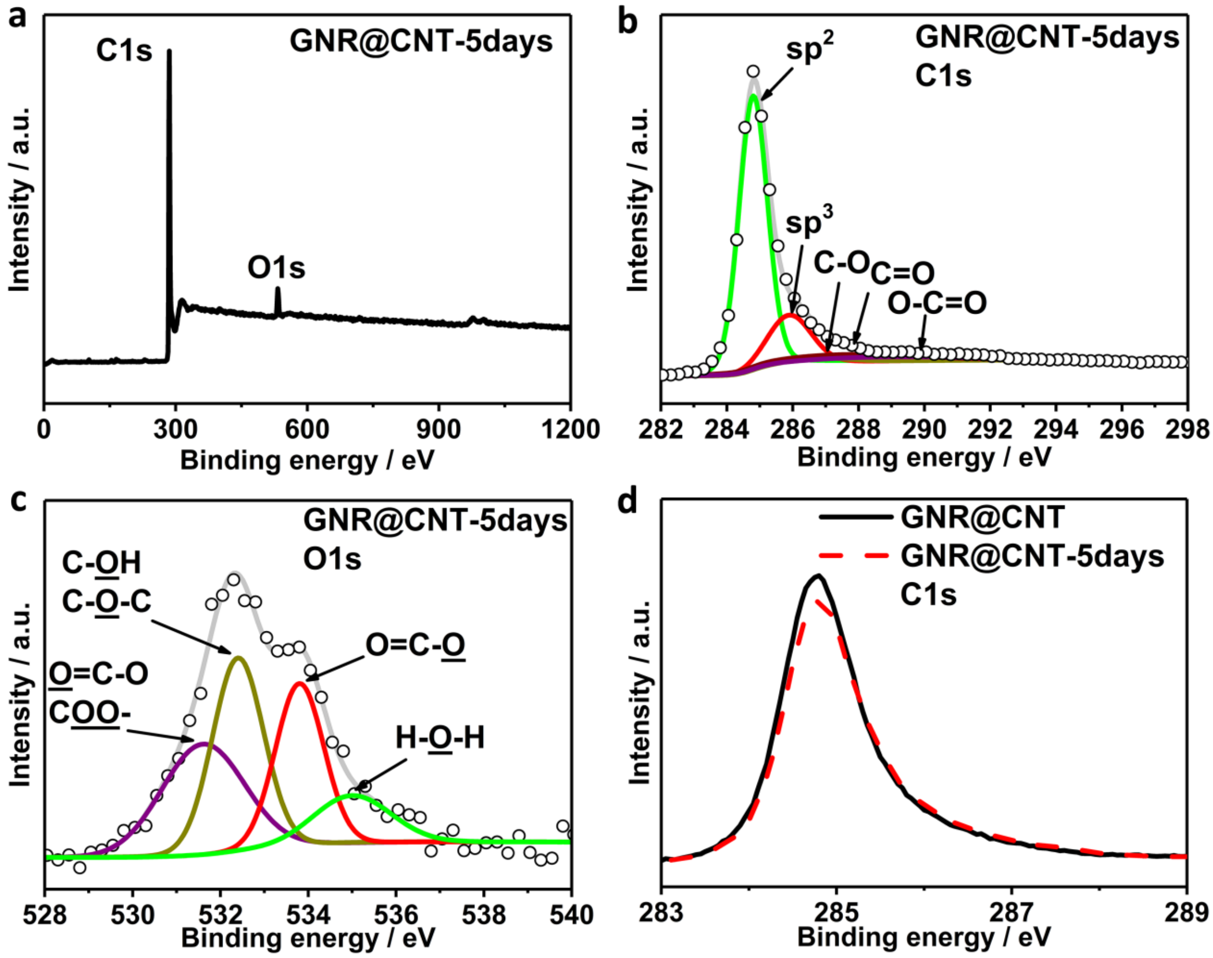


**Supplementary Figure 18** | **XPS spectra of GNR@CNT-5days.** (a) survey, (b)C1s, (c) O1s and (d) a comparison of C1s spectra between GNR@CNT and GNR@CNT-5days.

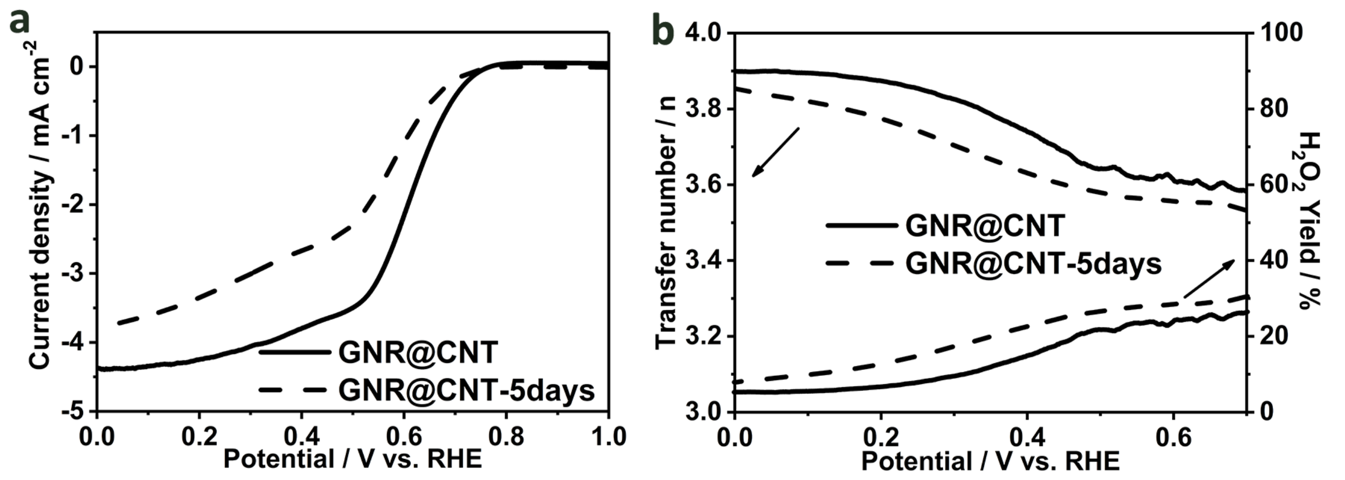


**Supplementary Figure 19 | Half-cell performance comparison after air oxidization.** (a) LSV curves and (b) electron transfer number and $H_2O_2$ yield of GNR@CNT (fresh) and GNR@CNT-5days (after exposed in air for 5 days) in $O_2$-saturated 0.5 M $H_2SO_4$ at 1600 rpm. Scan rate 10 mV $s^{-1}$.

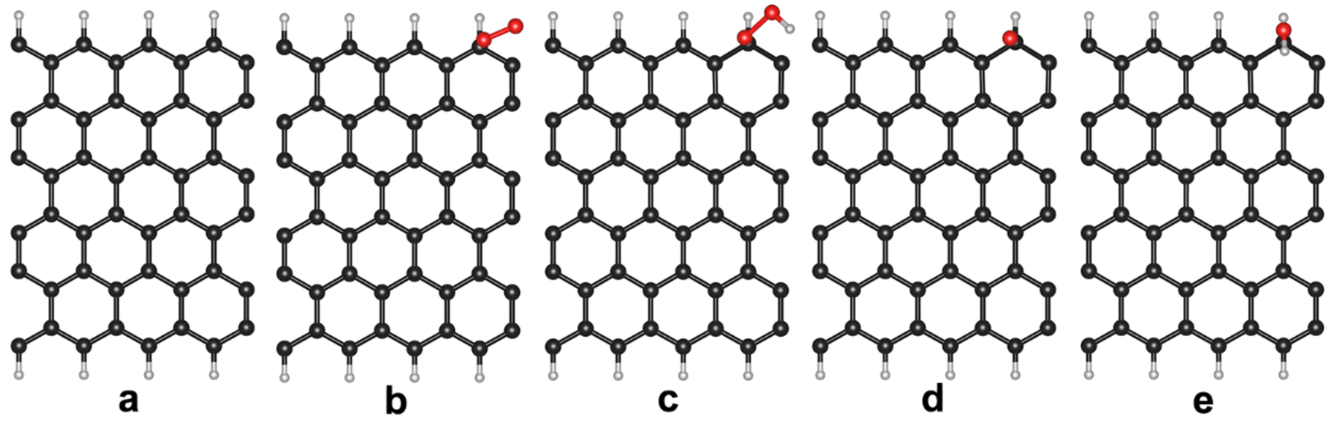


**Supplementary Figure 20 | Schematic representation of ORR process on the zigzag-edge of graphene nanoribbon.** (a) zigzag-edge graphene nanoribbons before ORR, (b) absorption of $O_2$ on a carbon at zigzag-edge, (c) adsorption of OOH after reaction with a proton, (d) adsorption of O after the release of a water molecule, and (e) adsorption of OH after reaction with a proton. Black, white, and red balls represent carbon, hydrogen, and oxygen atoms, respectively.

**Supplementary Table 1 | Relative elemental contents of GNR@CNT, N-GNR@CNT and GNR@CNT-5days extracted from the XPS results**

| Samples | C1s [at.%] | $sp^2/sp^3$ | N1s [at.%] | O 1s [at.%] | | | | |
|---|---|---|---|---|---|---|---|---|
| | | | | Total | C<u>OO</u>-<br><u>O</u>=C-O | C-<u>O</u>H<br>C-<u>O</u>-C | O=C-<u>O</u> | H-<u>O</u>-H |
| GNR@CNT | 97.73 | 8.77 | 0 | 2.27 | 0.73 | 1.05 | 0.41 | 0.07 |
| N-GNR@CNT | 94.56 | 4.0[a] | 3.09 | 2.35 | -- | -- | -- | -- |
| GNR@CNT-5days | 97.59 | 3.94 | 0 | 2.41 | 0.72 | 0.79 | 0.62 | 0.28 |

[a] C-N were contained in $sp^3$ for N-GNR@CNT.

**Supplementary Table 2 | ORR activities of carbon-based metal-free electrocatalyst from literatures measured by half-cell in 0.1 M KOH.**

| Catalyst | Catalyst loading (mg $cm^{-2}$) | Onset Potential (V vs. RHE) | Scan rate (mV $s^{-1}$) | Rotation Rate (rpm) | Current Density at 0.4 V (mA $cm^{-2}$) | Electron transfer number (n) | Reference |
|---|---|---|---|---|---|---|---|
| VA-NCNT | N.A. | 0.976 | 5 | 1400 | -3.90 | 3.9 | [1] |
| CNT | 0.255 | 0.846 | 10 | 1600 | -2.1 | 3.1 | [2] |
| N-porous carbon sheet | 0.2 | 0.956 | 5 | 1600 | -6.2 | 3.98 | [2] |
| N-graphene | 0.038 | 0.936 | 10 | 1600 | -3.06 | 3.3 | [3] |
| N-graphene/CNT | 0.05 | 0.866 | 20 | 1600 | -3 | 3.7 | [4] |
| N-graphitic arrays | 0.026 | 0.687 | 10 | 1600 | -5.7 | 3.89 | [5] |
| graphite-BM | 0.1 | 0.816 | 10 | 1600 | -1.75 | 3.8 | [6] |
| CNC700 | 0.1 | 0.876 | 10 | 2500 | -3.1 | 2.9 | [7] |
| GNR | 0.398 | 0.919 | 10 | 1600 | -3.8 | 3.88 | This work |
| N-GNR | 0.398 | 0.946 | 10 | 1600 | -5.2 | 3.95 | This work |
| GNR@CNT | 0.398 | 0.960 | 10 | 1600 | -4.8 | 3.94 | This work |
| N-GNR@CNT | 0.398 | 0.990 | 10 | 1600 | -5.1 | 3.96 | This work |

**Supplementary Table 3 | ORR activities of carbon-based metal-free electrocatalysts from literatures (measured in acidic electrolytes).**

| Catalyst | Electrolyte | Catalyst loading (mg cm$^{-2}$) | Onset Potential (V vs. RHE) | Scan rate (mV s$^{-1}$) | Rotation Rate (rpm) | Current Density at 0.3 V (mA cm$^{-2}$) | Electron transfer number (n) | Reference |
|---|---|---|---|---|---|---|---|---|
| N and P codoped mesoporous nanocarbon | 0.1 M $HClO_4$ | 0.450 | 0.83 | 5 | 1600 | 4.70 | 3.8 | [8] |
| N doped carbon nanotubes | 0.5 M $H_2SO_4$ | N.A. | 0.70 | 10 | 1600 | 1.73 | 3.52-3.92 | [9] |
| N doped carbon nanosheets | 0.5 M $H_2SO_4$ | 0.600 | 0.72 | 10 | 1600 | 4.91 | 3.67-3.91 | [10] |
| N doped carbon nanosheets | 0.5 M $H_2SO_4$ | 0.051 | 0.725 | 20 | 1600 | 2.17 | 3.90-3.98 | [11] |
| N doped mesoporous cabons | 0.5 M $H_2SO_4$ | 0.312 | 0.720 | 10 | 1600 | 3.95 | 3.48 | [12] |
| B and N codoped carbons | 0.5 M $H_2SO_4$ | 0.200 | 0.57 | 10 | 1500 | 0.68 | N.A. | [13] |
| GNR | 0.5 M $H_2SO_4$ | 0.398 | 0.52 | 10 | 1600 | 1.02 | 3.19-3.59 | This work |
| N-GNR | 0.5 M $H_2SO_4$ | 0.398 | 0.68 | 10 | 1600 | 3.81 | 3.90-3.96 | This work |
| GNR@CNT | 0.5 M $H_2SO_4$ | 0.398 | 0.76 | 10 | 1600 | 4.06 | 3.61-3.90 | This work |
| N-GNR@CNT | 0.5 M $H_2SO_4$ | 0.398 | 0.75 | 10 | 1600 | 3.17 | 3.72-3.92 | This work |

**Supplementary Table 4 | Gravimetric activities of various metal-free electrocatalysts compared with the N-GNR@CNT and GNR@CNT in PEM fuel cells.** All the data in the table have also been scaled by the electrode surface area.

| Materials | Current at 0.2 V (A $g^{-1}$) | Peak power density (W $g^{-1}$) | Catalyst loading (mg $cm^{-2}$) | $O_2$-$H_2$ absolute pressure (bars) | Cell temperature (℃) | Reference |
|---|---|---|---|---|---|---|
| Co-PPY-C | 725 | 156 | 0.8[a] | 2.5 | 80 | [14] |
| Fe/Phen/Z8 | 1500 | 233 | 3.9 | 1.5 | N.A. | [15] |
| (CM+PANI)-Fe-C | 900 | 225 | 4.0 | 2.5 | 80 | [16] |
| 20Co-NC-1100 | N.A. | 140 | 4.0 | 2.5 | 80 | [17] |
| Fe2-Z8-C | N.A. | 407 | 2.8 | 2.5 | 80 | [18] |
| (Fe,Co)/N-C | N.A. | 1272 | 0.77 | 2.5/1.5($O_2/H_2$) | 80 | [19] |
| bNGr | N.A. | 52 | 4 | 2.5 | N.A. | [20] |
| DMWNT–$H_2SO_4$-Ar900 | 297 | 60 | 1.85 | 3.5 | 90 | [21] |
| NG@MMT | 750 | 160 | 2 | N.A. | N.A. | [22] |
| VA-NCNT | 1550 | 320 | 0.16 | 2.5 | 80 | [23] |
| N-G-CNT+KB | 1500 | 300 | 0.5 | 2.5 | 80 | [23] |
| N-GNR@CNT+XC-72 | 2070/1950 | 420 / 380 | 0.25 / 0.5 | 2.5 | 80 | This work |
| GNR@CNT+XC-72 | 2400/1153 | 520 / 235 | 0.25 / 0.5 | 2.5 | 80 | This work |

[a] Here we regard Co-PPY as catalyst and substrate the quality of carbon black.

**Supplementary Table 5 | Open circuit voltage (OCV) of metal-free catalysts in PEMFC.**

| Materials | Catalyst loading / mg $cm^{-2}$ | OCV / $V_{RHE}$ |
|---|---|---|
| GNR | 0.25 | 0.749 |
| GNR@CNT | | 0.762 |
| N-GNR | | 0.793 |
| N-GNR@CNT | | 0.772 |
| GNR | 0.5 | 0.611 |
| GNR@CNT | | 0.776 |
| N-GNR | | 0.794 |
| N-GNR@CNT | | 0.722 |

## Supplementary references


1 Gong, K., Du, F., Xia, Z., Durstock, M. & Dai, L. Nitrogen-doped carbon nanotube arrays with high electrocatalytic activity for oxygen reduction. *Science* **323**, 760-764 (2009).

2 Zhong, H. X. *et al.* ZIF-8 derived graphene-based nitrogen-doped porous carbon sheets as highly efficient and durable oxygen reduction electrocatalysts. *Angew. Chem. Int. Ed.* **53**, 14235-14239 (2014).

3 Yang, S. *et al.* Efficient synthesis of heteroatom (N or S)-doped graphene based on ultrathin graphene oxide-porous silica sheets for oxygen reduction reactions. *Adv. Funct. Mater.* **22**, 3634-3640 (2012).

4 Chen, P., Xiao, T. Y., Qian, Y. H., Li, S. S. & Yu, S. H. A nitrogen-doped graphene/carbon nanotube nanocomposite with synergistically enhanced electrochemical activity. *Adv. Mater.* **25**, 3192-3196 (2013).

5 Liu, R., Wu, D., Feng, X. & Mullen, K. Nitrogen-doped ordered mesoporous graphitic arrays with high electrocatalytic activity for oxygen reduction. *Angew. Chem. Int. Ed.* **49**, 2565-2569 (2010).

6 Shen, A. *et al.* Oxygen reduction reaction in a droplet on graphite: direct evidence that the edge is more active than the basal plane. *Angew. Chem. Int. Ed.* **53**, 10804-10808 (2014).

7 Jiang, Y. *et al.* Significant contribution of intrinsic carbon defects to oxygen reduction activity. *ACS Catal.* **5**, 6707-6712 (2015).

8 Zhang, J., Zhao, Z., Xia, Z. & Dai, L. A metal-free bifunctional electrocatalyst for oxygen reduction and oxygen evolution reactions. *Nat. Nanotechnol.* **10**, 444-452 (2015).

9 Yu, D., Zhang, Q. & Dai, L. Highly efficient metal-free growth of nitrogen-doped single-walled carbon nanotubes on plasma-etched substrates for oxygen reduction. *J. Am. Chem. Soc.* **132**, 15127-15129 (2010).

10 Wei, W. *et al.* Nitrogen-doped carbon nanosheets with size-defined mesopores as highly efficient metal-free catalyst for the oxygen reduction reaction. *Angew. Chem. Int. Ed.* **53**, 1570-1574 (2014).

11 Chen, P. *et al.* Nitrogen-doped nanoporous carbon nanosheets derived from plant biomass: an efficient catalyst for oxygen reduction reaction. *Energy Environ. Sci.* **7**, 4095-4103 (2014).

12 Wang, X. *et al.* Ammonia-treated ordered mesoporous carbons as catalytic materials for oxygen reduction reaction. *Chem. Mater.* **22**, 2178-2180 (2010).

13 Ozaki, J.-I., Kimura, N., Anahara, T. & Oya, A. Preparation and oxygen reduction activity of BN-doped carbons. *Carbon* **45**, 1847-1853 (2007).

14 Bashyam, R. & Zelenay, P. A class of non-precious metal composite catalysts for fuel cells. *Nature* **443**, 63-66 (2006).

15 Proietti, E. *et al.* Iron-based cathode catalyst with enhanced power density in polymer electrolyte membrane fuel cells. *Nat. Commun.* **2**, 416 (2011).

16 Chung, H. T. *et al.* Direct atomic-level insight into the active sites of a high-performance PGM-free ORR catalyst. *Science* **357**, 479-484 (2017).

17 Wang, X. X. *et al.* Nitrogen-coordinated single cobalt atom catalysts for oxygen reduction in proton exchange membrane fuel cells. *Adv. Mater.* **30**, 1706758 (2018).

18 Liu, Q., Liu, X., Zheng, L. & Shui, J. The solid-phase synthesis of an Fe-N-C electrocatalyst for high-power proton-exchange membrane fuel cells. *Angew. Chem. Int. Ed.* **57**, 1204-1208 (2018).

19 Wang, J. *et al.* Design of N-coordinated dual-metal sites: a stable and active Pt-free catalyst for acidic oxygen reduction reaction. *J. Am. Chem. Soc.* **139**, 17281-17284 (2017).

20 Choi, C. H. *et al.* Long-range electron transfer over graphene-based catalyst for high-performing oxygen reduction reactions: importance of size, N-doping, and metallic impurities. *J. Am. Chem. Soc.* **136**, 9070-9077 (2014).

21 Waki, K. *et al.* Non-nitrogen doped and non-metal oxygen reduction electrocatalysts based on carbon nanotubes: mechanism and origin of ORR activity. *Energy Environ. Sci.* **7**, 1950-1958 (2014).

22 Ding, W. *et al.* Space-confinement-induced synthesis of pyridinic- and pyrrolic-nitrogen-doped graphene for the catalysis of oxygen reduction. *Angew. Chem. Int. Ed.* **52**, 11755-11759 (2013).

23 Shui, J., Wang, M., Du, F. & Dai, L. N-doped carbon nanomaterials are durable catalysts for oxygen reduction reaction in acidic fuel cells. *Sci. Adv.* **1**, e1400129 (2015).
